# The Global Hypophosphatasia Registry: lessons learned from a decade of real-world data

**DOI:** 10.1186/s13023-025-04129-w

**Published:** 2025-11-24

**Authors:** Priya S. Kishnani, Lothar Seefried, Keiichi Ozono, Gabriel Ángel Martos-Moreno, Cheryl Rockman-Greenberg, Deborah Fowler, Luke K. Burke, William R. Mowrey, Eric T. Rush, Peter R. Ebeling, Wolfgang Högler, Agnès Linglart, Shona Fang, Anna Petryk, Kathryn M. Dahir

**Affiliations:** 1 Duke University Medical Center, Durham, NC USA; 2 University of Würzburg, Würzburg, Germany; 3 Iseikai International General Hospital, Osaka, Japan; 4 IIS La Princesa, Hospital Infantil Universitario Niño Jesús, Universidad Autónoma de Madrid, ISCIII, CIBERobn, Madrid Spain; 5 University of Manitoba, Winnipeg, MB Canada; 6 Soft Bones, Boonton, NJ USA; 7 Global Scientific Communications, Alexion, AstraZeneca Rare Disease, Barcelona, Spain; 8 Bioinformatics, Alexion, AstraZeneca Rare Disease, Boston, MA USA; 9 Children’s Mercy Kansas City, Kansas City, MO USA; 10 University of Missouri – Kansas City School of Medicine, Kansas City, MO USA; 11 School of Clinical Sciences, Monash University, Clayton, VIC Australia; 12 Department of Pediatrics and Adolescent Medicine, Johannes Kepler University Linz, Linz, Austria; 13 Department of Metabolism and Systems Science, School of Medical Sciences, College of Medicine and Health, University of Birmingham, Birmingham, UK; 14 Paris-Saclay University, AP-HP and Inserm, Kremlin-Bicêtre, France; 15 Epidemiology and Real World Science, Alexion, AstraZeneca Rare Disease, Boston, MA USA; 16 Global Medical Affairs, Alexion, AstraZeneca Rare Disease, Boston, MA USA; 17 Vanderbilt University Medical Center, Nashville, TN USA; 18 YT and Alice Chen Pediatric Genetics and Genomics Research Center, Molecular Genetics and Microbiology, Duke University Medical Center, 905 Lasalle Street, GSRB 1, 4th Floor, Room 4010, Durham, NC 27710 USA

## Abstract

**Introduction:**

Hypophosphatasia (HPP) is an inherited, metabolic, rare disease characterized by a high level of clinical heterogeneity. In response to this robust heterogeneity, the Global HPP Registry was formed to characterize the types of manifestations that patients may experience, as well as to compile information on genetic underpinnings of the disease, overall impact on patient quality of life, and safety and effectiveness of enzyme replacement therapy. The objective of this review was to synthesize key learnings gained from the Global HPP Registry, which is now in its tenth year of enrolling patients.

**Methods:**

Registry data were analyzed to provide up-to-date information on age at diagnosis of HPP and alkaline phosphatase substrate testing. Published articles and abstracts reporting results from the registry were reviewed and summarized.

**Results:**

Analyses showed peaks in age at diagnosis of HPP in early childhood and middle adulthood. Pyridoxal 5′-phosphate testing was performed in 18% to 61% of registry patients across geographic regions, and phosphoethanolamine testing was performed in 5% to 48% of registry patients. Published reports demonstrate that nonskeletal manifestations of HPP are an important disease feature that can affect functional outcomes. The review also reports recent findings on the genetics of HPP across a broad patient population, including heterozygous patients, and integrated literature showing that patients with HPP can have high levels of disease burden regardless of whether they present with overt skeletal manifestations or if the disease first presents in childhood or adulthood. Based on the collective findings of this review, an updated classification system for patients with HPP is proposed that incorporates a more recent understanding of the spectrum of this condition. Outcomes showing the effectiveness of enzyme replacement therapy among children and adults treated in a real-world setting are also included.

**Conclusions:**

In summary, learnings from the past decade of the registry have improved the overall understanding of HPP in a wide patient population and may play an important role in improving disease recognition and diagnosis.

**Supplementary Information:**

The online version contains supplementary material available at 10.1186/s13023-025-04129-w.

## Introduction

Hypophosphatasia (HPP) is a rare inherited, metabolic disease caused by variants in the gene *ALPL*, which encodes tissue-nonspecific alkaline phosphatase (ALP) [1]. The carrier frequency of *ALPL* variants is estimated to be 1:187 to 1:274, with higher carrier frequency in founder populations [2–4]. Pathogenic *ALPL* variant(s) are inherited in an autosomal dominant or autosomal recessive manner with variable penetrance and can lead to deficient age- and sex-adjusted ALP enzyme activity and subsequent deficiency of its products and extracellular accumulation of its substrates [4–6]. These substrates include inorganic pyrophosphate (PPi, an inhibitor of bone mineralization), pyridoxal 5′-phosphate (PLP, the circulating form of vitamin B_6_), and phosphoethanolamine (PEA). ALP also cleaves several other compounds including adenosine triphosphate, adenosine diphosphate, and adenosine monophosphate [1, 7].

HPP is characterized by diverse clinical, radiological, and biochemical features that broadly include skeletal manifestations, such as rickets/osteomalacia, dental abnormalities, fractures, pseudofractures, and craniosynostosis, as well as nonskeletal manifestations, such as muscle weakness or stiffness, constitutional symptoms, fatigue, neurological problems, nephrocalcinosis, ectopic calcifications, and pain [8]. These manifestations can vary throughout a patient's lifespan [8, 9]. Infants with HPP may experience potentially lethal manifestations, including respiratory failure, discussed in greater detail below [8]. Clinical manifestations of HPP cause poor quality of life, impaired mobility and recurrent or poorly healing fractures, symptoms that can accumulate and change over time.

The prevalence of perinatal/infantile HPP is estimated to be between 1 in 100,000 and 1 in 500,000 births based on data from France, Canada, and Japan [10–12]. In one report, HPP prevalence beyond perinatal/infantile HPP was estimated at 1 in 2,430 for “moderate” HPP and 1 in 508 for “mild” HPP, although these estimates and the definitions of moderate and mild HPP should be interpreted cautiously as they are based on the frequency of specific *ALPL* variants and lack clinical validation [3]. Furthermore, prevalence estimates from that report were calculated based on a defined variant penetrance of 50%, which does not align with the reality of *ALPL* variant penetrance [3, 13, 14]. An analysis of Systematized Nomenclature of Medicine–Clinical Terms, clinical notes, and prescriptions for asfotase alfa enzyme replacement therapy in US electronic health records estimated a diagnosed HPP prevalence of 2.8 in 100,000 in the United States, although this may be an underestimate [15].

Successful diagnosis of HPP is challenged by its status as a rare disease and the complex array of clinical and genetic features seen across affected patients [16]. Furthermore, the current HPP classification may not accurately reflect underlying disease pathophysiology [17]. The Global HPP Registry (NCT02306720, EUPAS13514; sponsored by Alexion, AstraZeneca Rare Disease) represents the largest observational study of patients with HPP, comprising over 1,500 patients across 12 countries, and is reviewed by HPP experts across different disciplines to better understand and advance global knowledge of the disease. Now in its tenth year, the Global HPP Registry has provided real-world data that have addressed pressing scientific questions, significantly evolved how we think about HPP, and continuously contributed to improved patient care. Here, we review 10 years of learnings, including an expanded understanding of the disease and its diagnosis; the burden in children and adults beyond direct impacts on the skeletal system; the disease burden in patients with autosomal dominant or autosomal recessive inheritance; the importance of physical function, pain, and quality-of-life assessments; and treatment goals.

## Registry learnings on HPP diagnosis

### Diagnostic delays

The variable presentation of patients with HPP, its rare occurrence, and the lack of knowledge on rare bone disease outside tertiary care complicates timely detection of disease and can lead to significant diagnostic delays. This is especially true among adults with suspected HPP, who generally have a broader range of nonspecific clinical features compared with children. The median (range) diagnostic delay was 24.5 (0–46.3) years among adults in the registry who experienced first symptoms in childhood [18]. In comparison, the median (range) diagnostic delay was 8.4 months (0–10.7 years) among children in the registry who first presented at 1 to < 18 years of age [18]. These diagnostic delays occurred despite approximately half of patients in this analysis having a family history of disease, underscoring the importance of performing a pedigree analysis and evaluating at-risk family members once probands are identified [18].

### Age and sex at diagnosis

**Fig. 1:**
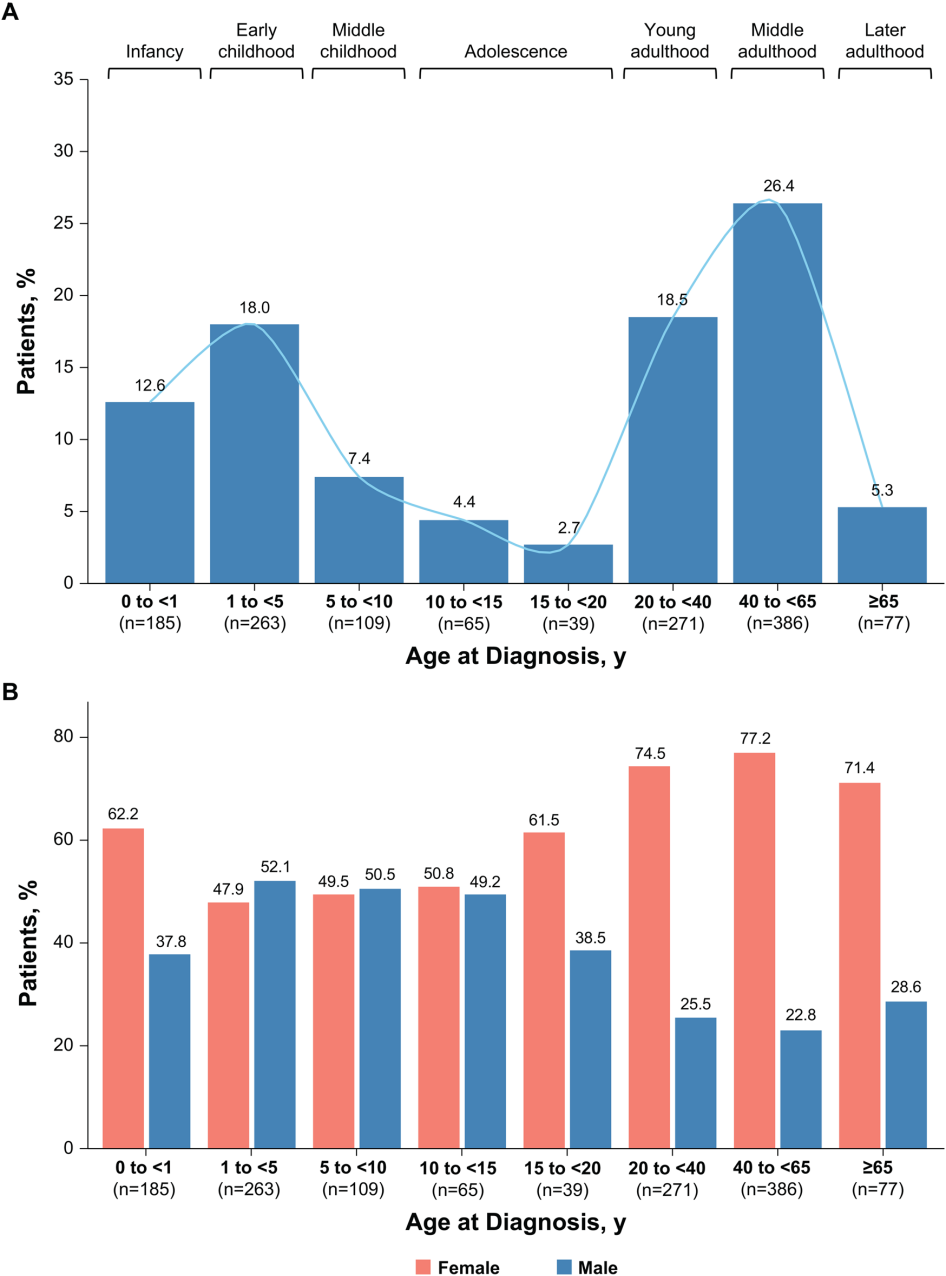
Proportion of patients diagnosed with HPP stratified by (**A**) age and (**B**) sex, HPP, hypophosphatasia

Peak age groups at time of HPP diagnosis are in infancy, early childhood, and in middle adulthood. A 2019 analysis of data from patients in the registry showed a nonuniform distribution of age at diagnosis, with peaks at ages 0 to < 6 months, ages 2 to < 10 years, and ages ≥ 50 years [18]. An updated analysis of registry data (data cut: December 2024; Supplementary Methods and Figure S1 in Additional File 1) also showed nonuniform distribution, with peaks in infancy (13% of all diagnoses), early childhood (18%), and middle adulthood (26%; Fig. 1A).

Sex distribution by age at diagnosis was similarly nonuniform among patients enrolled in the registry. Male and female patients with HPP were represented in approximately equal proportions from early childhood to early adolescence (Fig. 1B). However, from late adolescence onward, female sex was clearly predominant among patients in the registry. This trend continued throughout life, with women accounting for approximately 75% of diagnoses in adulthood (Fig. 1B).

The reasons for higher rate of females versus males in the registry are not currently clear. A similar enrichment of female patients has been observed in hypermobile Ehlers-Danlos syndrome beginning at puberty, which may be driven by hormonal changes [19]. It is unknown whether hormonal effects could underlie sex differences in HPP. Other hypotheses potentially underlying sex differences in HPP may include (1) screening and prevention programs for osteoporosis that could lead to detection of low ALP activity or HPP among postmenopausal women; (2) women who seek care for osteoporosis or fibromyalgia (both more common among women than men [20, 21]) may be diagnosed with HPP if an *ALPL* variant and biochemical signature of disease (i.e., low ALP activity and accumulation of ALP substrates [22]) are detected; (3) higher levels of health awareness among women, which drive them to seek care for medical problems [23]; and (4) higher rates of registry enrollment among women. Each of these potential reasons is speculative and requires further research.

### Measurement of ALP substrates in diagnosis

**Fig. 2:**
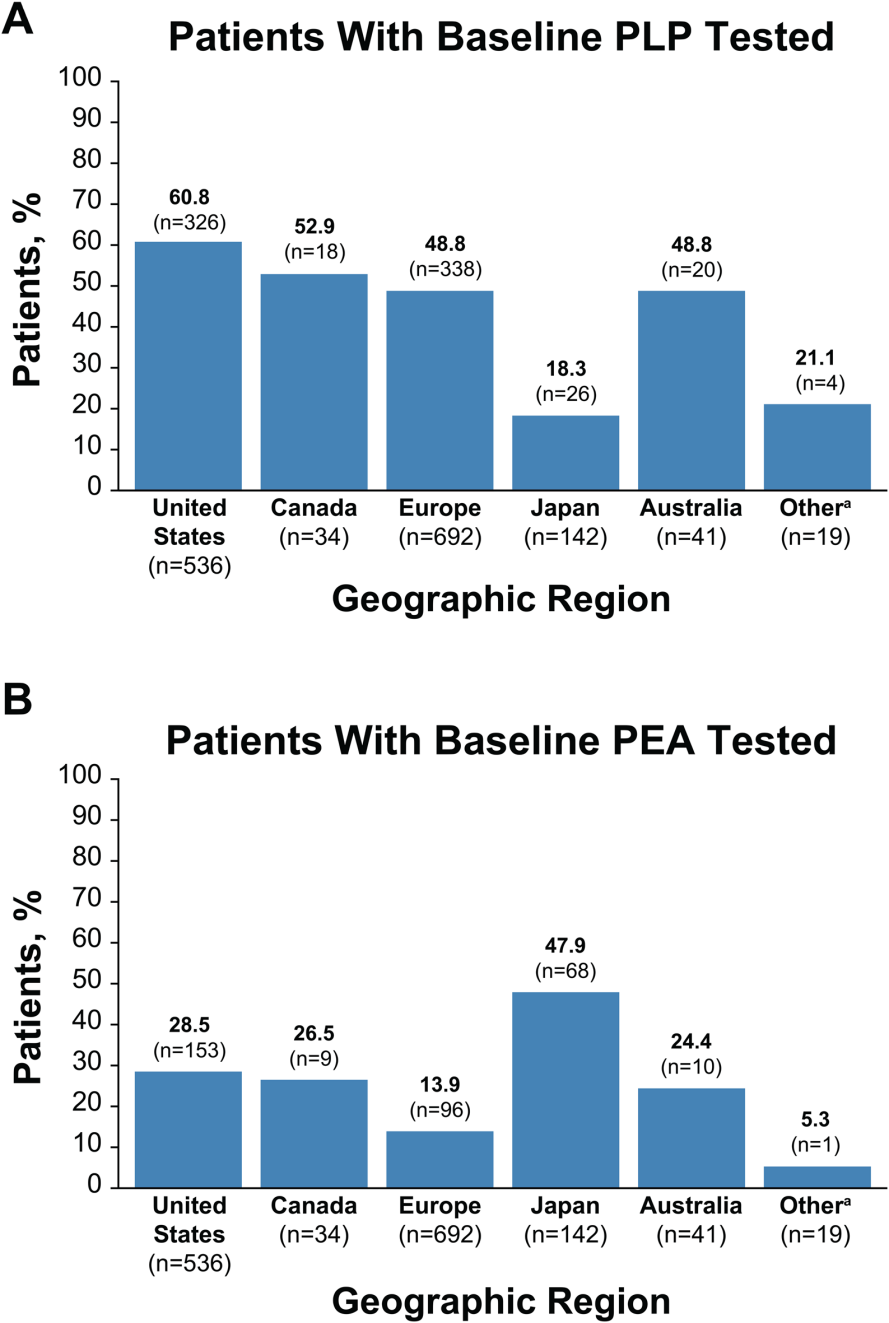
Proportion of patients with (**A**) PLP and (**B**) PEA testing by region. Baseline refers to testing performed in asfotase alfa treatment-naive patients. ^a^Other countries include Israel, Russia, Saudi Arabia, Taiwan, and Turkey. PEA, phosphoethanolamine; PLP, pyridoxal 5'-phosphate

Measurement of ALP substrates is a useful approach to support a diagnosis of HPP [5, 24–26]. Biochemical assessments of plasma PLP and urinary PEA are both commercially available (in contrast to PPi, which is typically only used in research settings), making them particularly relevant laboratory assessments in individuals with suspected HPP [8]. Despite the utility of substrate analysis, actual use of this approach for patients in the registry varies by region (Fig. 2A, B). Across all regions captured in the registry, PLP is more commonly measured than PEA, possibly due to broader testing ability. Half of patients had baseline PLP testing, which was most common among patients in the United States (61%) and least common among patients in Japan (18%) and the region referred to as “Other” (21%; included Israel, Russia, Saudi Arabia, Taiwan, and Turkey). Almost a quarter (23%) of patients globally had PEA testing at baseline. PEA testing was most common among patients in Japan (48%) followed by the United States (29%). Only 14% of patients in Europe had PEA testing at baseline. The apparent preference for PEA testing in Japan likely reflects the high commercial availability of urinary PEA assessment and lack of insurance coverage for PLP assessment in Japan; insurance coverage of urinary PEA assessment is available in Japan [27].

**Fig. 3:**
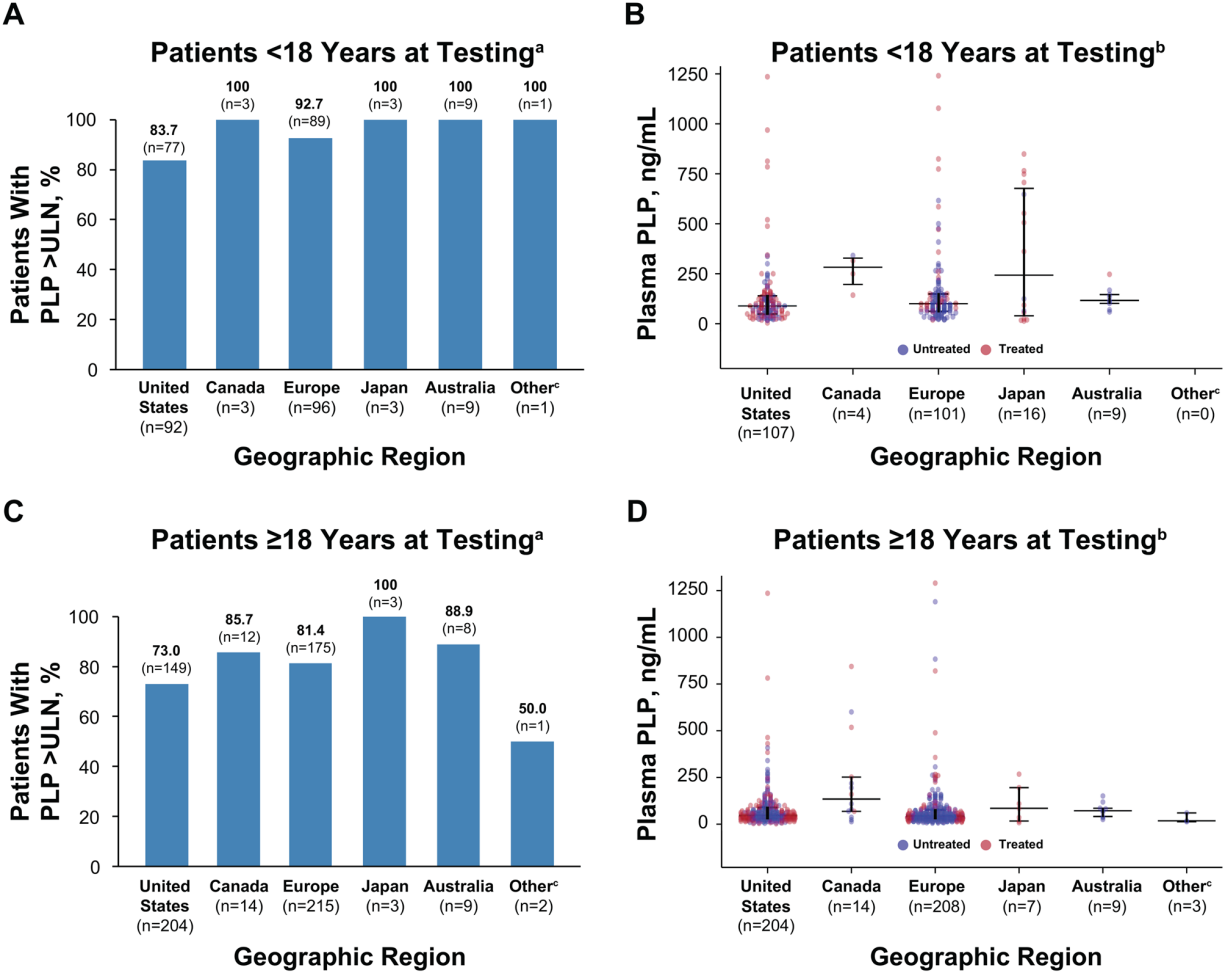
(**A**) Proportion of children with plasma PLP concentrations above the upper limit of normal and (**B**) Plasma PLP concentrations in children. (**C**) Proportion of adults with plasma PLP concentrations above the upper limit of normal and (**D**) Plasma PLP concentrations in adults. ^a^Included patients with nonmissing site-entered upper limit of normal data. ^b^Included only values that were entered in ng/mL or could be converted to ng/mL. PLP values > 1500 ng/mL were excluded as outliers. ^c^Other countries include Israel, Russia, Saudi Arabia, Taiwan, and Turkey. PLP, pyridoxal 5’-phosphate; ULN, upper limit of normal

Among treatment-naive patients in the registry who had available PLP data, most had plasma concentrations that exceeded the upper limit of normal. Among children who had PLP data, 89% showed PLP concentrations above the upper limit of normal, with minor differences by geographic region (range: 84%–100% of patients; Fig. 3A). Across all geographic regions assessed, the median PLP concentration in untreated children ranged from 89.0 (United States) to 282.5 ng/mL (Canada, Fig. 3B). Similarly, 78% of all adults had PLP concentrations above the upper limit of normal (Fig. 3C). The percentage of adults with PLP concentrations above the upper limit of normal was lowest in the Other region (Israel, Russia, Saudi Arabia, Taiwan, and Turkey), although only 2 patients from this region had available data (Fig. 3C). The median PLP concentration among untreated adults from all geographic regions ranged from 17.7 (Other region) to 134.3 ng/mL (Canada, Fig. 3D).

**Fig. 4:**
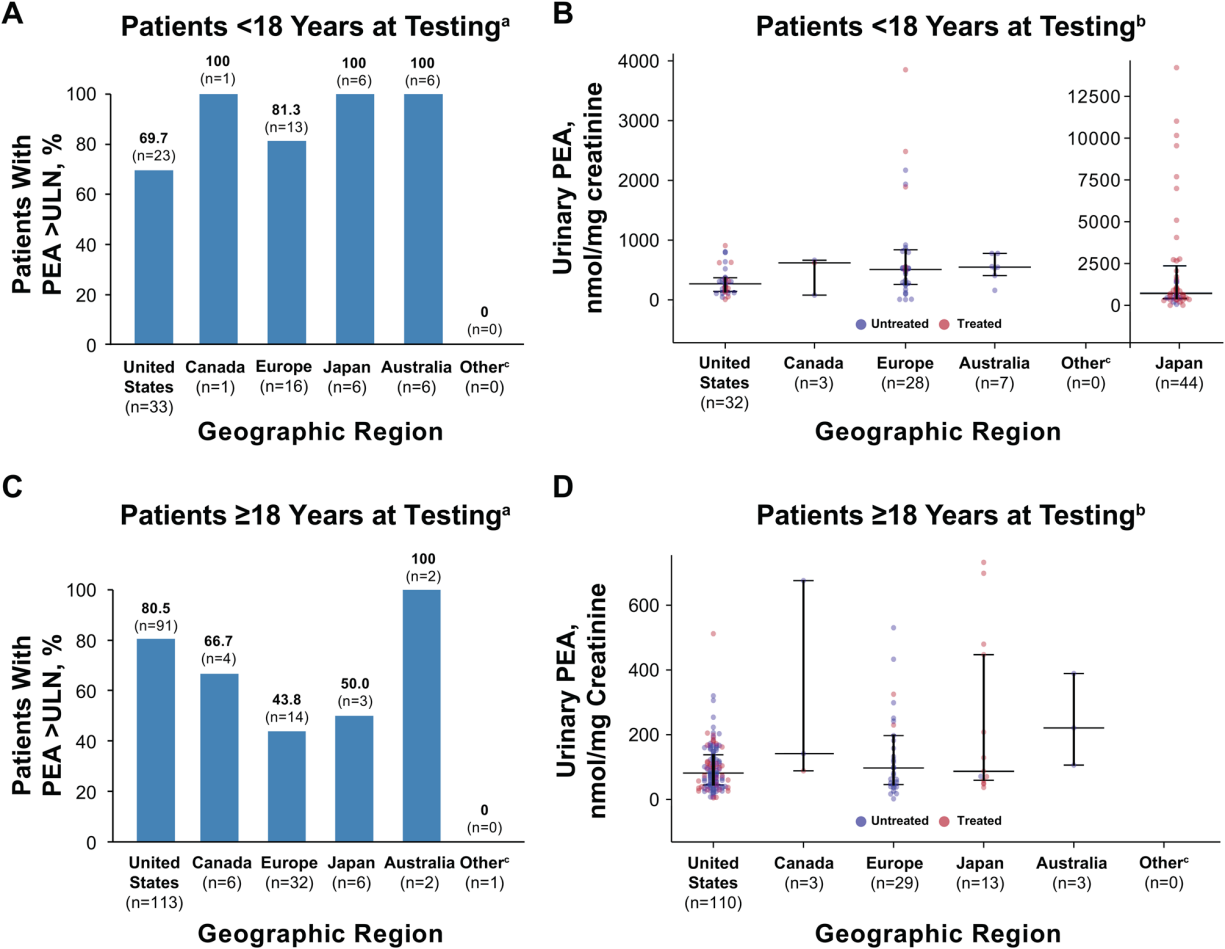
(**A**) Proportion of children with urinary PEA concentrations above the upper limit of normal and (**B**) Urinary PEA concentrations in children. (**C**) Proportion of adults with urinary PEA concentrations above the upper limit of normal and (**D**) Urinary PEA concentrations in adults. ^a^Included patients with nonmissing site-entered ULN data. ^b^Included only values that were entered in nmol/mg creatinine or could be converted to nmol/mg creatinine. ^c^Other countries include Israel, Russia, Saudi Arabia, Taiwan, and Turkey. PEA, phosphoethanolamine; ULN, upper limit of normal

Among patients with PEA data, most untreated children (79%) and adults (71%) with HPP had PEA levels above the upper limit of normal except for adults in Europe, among whom only 44% had concentrations above the upper limit of normal (Fig. 4A, C). Median urinary PEA concentration ranged from 268.0 (United States) to 723.6 nmol/mg creatinine (Japan) in untreated children and from 81.5 (United States) to 221.0 nmol/mg creatinine (Australia) in untreated adults in the registry (Fig. 4B, D).

**Fig. 5:**
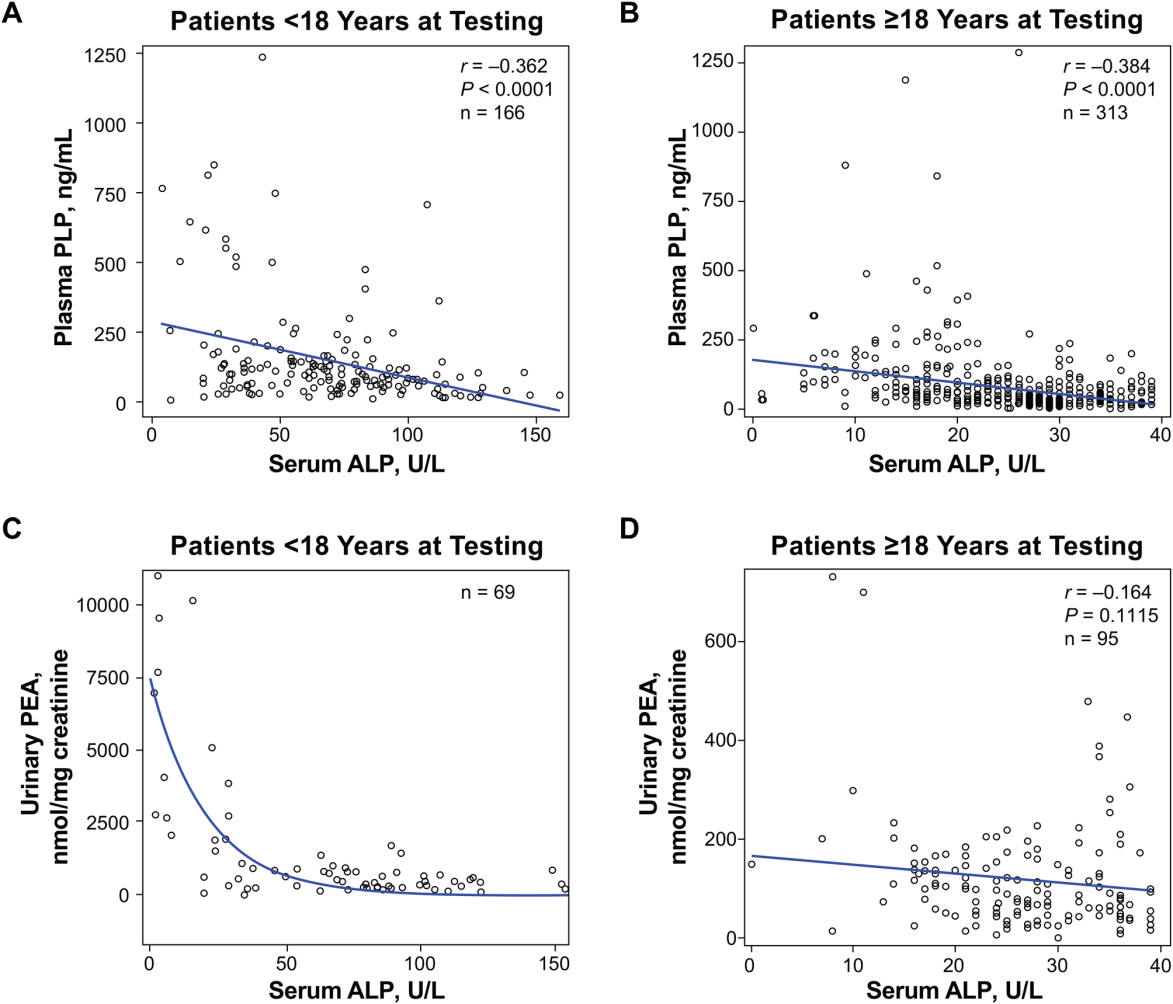
Spearman correlations between serum ALP and plasma PLP in (**A**) children and (**B**) adults. Exponential relationship (**C**) between serum ALP and urinary PEA in children. Spearman correlation (**D**) between serum ALP and urinary PEA in adults. ALP, alkaline phosphatase; PEA, phosphoethanolamine; PLP, pyridoxal 5′-phosphate

Spearman correlations between ALP and its substrates were analyzed for children and adults with low age- and sex-adjusted ALP activity, defined here as < 40 U/L in adults or < 160 U/L in children. There was a weak inverse correlation between ALP activity and PLP concentrations in all patients (children: *r* = − 0.36, *P* < 0.0001; adults: *r* = − 0.38, *P* < 0.0001; Fig. 5A, B). Thus, PLP is a particularly useful metric when ALP activity is low. There was an exponential relationship between ALP activity and urinary PEA concentration among children (Fig. 5C), while no correlation was observed among adults (*r* = − 0.16, *P* = 0.1115; Fig. 5D).

Real-world data collected for analysis in the registry are limited by lack of standard collection methods and potential artifacts related to dietary or supplemental intake of vitamin B_6_ (specifically for assessment of PLP). PLP is photosensitive and unstable in plasma samples stored at − 20 °C or warmer [28, 29]. Plasma samples are stable at − 80 °C for up to 10 days of assessment, and use of amber vials to protect specimens from light and either immediate processing or storage of samples in ultra-low temperature freezers is warranted [30, 31]. Blood is often collected from patients after an overnight fast for measurement of PLP [32, 33]. Guidance on HPP diagnostic criteria published by an international working group in 2024 recommends that patients stop taking any supplements or fortified foods containing high levels of vitamin B_6_ (including sports or energy drinks) prior to PLP measurement [5]. Notably, assessment of PLP among patients treated with enzyme replacement therapy is typically not feasible in routine clinical practice since this would require use of a potent ALP inhibitor such as levamisole to avoid skewed results due to persistent recombinant enzyme activity in test tubes [8]. To assess urinary PEA, research efforts are needed to determine any cold storage requirements. Further, more information is needed to determine whether random urine samples are sufficient for assessing PEA and to understand the effect of overnight fasting on PEA measured in first void specimens [34].

### HPP genetics

Before registry data were analyzed, information on the role of specific genetic variants in disease development was limited to case series identifying regional founder variants (e.g., c.1001G >A or c.1559delT) or case studies reporting single variants [35, 36]. Several recently published guidelines have emphasized the importance of genetic testing to confirm HPP diagnosis [5, 24–26, 37], and data from the registry have provided new insights into the many *ALPL* gene variants in patients with HPP.

A particularly striking finding in 814 patients from the HPP registry is that 75% carried a single *ALPL* variant while only 25% carried ≥ 2 variants, definitively supporting that a heterozygous variant can be sufficient to cause HPP [13]. Phasing data were not available to assess if multiple variants were inherited in *cis* or in *trans*. Among heterozygotes with a variant previously tested for dominant-negative activity, only 42% had a variant with evidence of a dominant-negative effect. This finding highlights the need to elucidate other mechanisms contributing to disease manifestations in heterozygotes, such as additional pathogenic variants in noncoding regions not covered by sequencing, *cis*- or *trans*-acting genetic modifiers, environmental exposures, modifying genes, or other factors [38].

In patients with disease onset before 6 months of age, the majority (72%) had 2 *ALPL* variants detected, while the remaining 28% had 1 variant reported [13]. In patients with disease onset after 6 months, the majority (83%) had 1 reported variant [13]. These data indicate a predominance of homozygous or compound heterozygous variants in patients with disease onset before 6 months [13]. Across all regions assessed, most (69%–86%) patients had 1 *ALPL* variant, except in Japan where 31% of patients had 1 variant and the remaining 69% had 2 variants. Further efforts to explore genetics in HPP are underway to cross-reference data from the registry with the JKU *ALPL* gene variant database (https://alplmutationdatabase.jku.at/) to tie phenotypes to genotypes and improve variant interpretations [26].

### Broader understanding of clinical profiles of pediatric and adult patients with HPP

Findings from the registry have substantially expanded our understanding of the clinical profile of patients with HPP. In 1948, the first reported case study of HPP, then called Rathbun’s syndrome, described an infant who presented with skeletal abnormalities, poor growth, and seizures [39]. Given the role of tissue-nonspecific ALP in regulating bone mineralization through cleavage of PPi [7], much of the research in HPP has focused on skeletal manifestations. Despite this, early observations from the registry showed that only 44% of children and 43% of adults reported a history of overt skeletal manifestations, indicating that HPP can cause relevant clinical burden without obvious skeletal symptoms [18, 40]. Thus, data from patients who survive past infancy demonstrate that HPP is a multisystemic disorder. Other inherited disorders, including cystic fibrosis and Duchenne’s muscular dystrophy, have similarly demonstrated multisystemic manifestations after development of treatment strategies that significantly bolstered disease survival [41, 42].

In recognition of the wide variety of manifestations experienced by patients with HPP, clinical signs and symptoms are classified in the registry into 9 major categories: skeletal, dental, pain, muscular, rheumatic, neurologic, constitutional/metabolic, renal, and respiratory. While patients of all ages may have (or have a history of) these types of presentations, the prevalence of some features varies by patient age [18].

Among children who went on to receive asfotase alfa treatment, approximately one third had documented rickets, muscle weakness, gross motor delay, early loss of primary teeth, and/or hypercalcemia/hypercalciuria/hyperphosphatemia at baseline [43]. A quarter of these children reported pain, including 20% who reported chronic bone pain. At baseline (i.e., before treatment with enzyme replacement therapy), most children in the registry had height within the normal range, with short stature (defined as height < 3rd percentile) recorded for 17% of patients aged < 2 years and 20% of patients aged ≥ 2 years [44]. Impaired growth velocity resulting in short stature among patients aged < 2 years indicates that HPP can impair growth plate activity during infancy, as expected given previous histological evidence [44, 45]. A broad range of clinical manifestations occurred in those above and below the third percentile for height, suggesting that height alone may not accurately reflect HPP disease burden [44].

Among children with HPP, a key finding from registry analysis is that patients with disease onset before 6 months of age have unique life-threatening clinical characteristics compared with patients with disease onset after 6 months of age. In particular, respiratory failure and vitamin B_6_-responsive seizures are significantly more common in this youngest subset of patients, and both of these manifestations can be life-threatening. Historical data prior to the inception of the registry showed that patients who developed manifestations before 6 months of age had a 5-year survival rate of only 27% [46]. These data, combined with the observation that most patients in this age group have ≥ 2 *ALPL* variants [13], collectively suggest that patients who first present before 6 months of age reflect a distinct clinical phenotype.

The registry has significantly improved our understanding of HPP presentation in adults, providing a robust accumulation of data in this underrecognized population. As with children, adults with HPP present with a wide range of clinical features, including skeletal and nonskeletal manifestations [40, 47]. Among adults with any age of disease onset, 62% had a history of fracture or pseudofracture [40]. Among adults with pediatric-onset disease who went on to receive asfotase alfa treatment, commonly reported baseline manifestations included chronic bone pain (66% of patients), generalized body pain (58%), chronic muscle pain (47%), early loss of primary teeth (47%), fatigue (47%), muscle weakness (37%), and abnormal gait (29%) [47].

In sum, data from the registry demonstrate that there are no clinical manifestations experienced by all patients with HPP, although some form of pain is often reported. This finding has broadened the definition of how HPP can present in patients, including co-presentation of skeletal and nonskeletal manifestations or complete clinical absence of overt skeletal manifestations. The heterogeneity of clinical features of HPP is a testament to the challenge of its clinical diagnosis and underscores the importance of combined clinical, biochemical, and genetic evaluation to support diagnosis in patients of all ages.

### Disease burden in HPP

Data from the registry have elucidated the substantial disease burden and impaired quality of life among patients with HPP. Nearly 40% of adults enrolled in the registry reported having experienced at least 5 clinical signs and symptoms of HPP, and 57% reported at least 3 body systems affected (e.g., dental, skeletal, muscular) [40]. Disease burden among patients with HPP has been observed in multiple analyses regardless of (1) age at symptom onset (pediatric vs. adult) [48], (2) symptomatology (primary skeletal vs. nonskeletal) [49], or (3) number of *ALPL* variants (1 or ≥ 2) [49].

One analysis of data from adults in the registry with pediatric-onset HPP (first symptom presentation at < 18 years of age) versus adult-onset HPP found no significant differences in pain severity and interference, use of assistive devices or home modifications, or quality of life) [40]. Among all adults in the analysis, 17% used an assistive device or needed home modification [40].

A similar analysis of data from adults in the registry compared outcomes among patients who reported skeletal manifestations with outcomes among patients who reported only pain or muscular manifestations [48]. There were no differences in self-assessed disability, distance walked on the 6-Minute Walk Test (6MWT), or quality of life between the 2 groups, although a greater percentage of patients with skeletal versus without skeletal manifestations used mobility aids (29% vs. 10%) [48].

In another analysis of registry data to determine disease burden, outcomes were compared between patients with 1 versus ≥ 2 *ALPL* variants who presented with manifestations of HPP after 6 months of age [49]. No statistically significant differences were reported in pain (assessed with the Brief Pain Inventory–Short Form), disability (Health Assessment Questionnaire Disability Index), or quality of life (Pediatric Quality of Life Inventory for children and Short Form-36 Health Survey version 2 for adults) between patients with 1 versus ≥ 2 variants. Distance walked on the 6MWT was similar between variant groups for children but significantly worse for adults with ≥ 2 *ALPL* variants, as assessed by 95% confidence intervals (although only 8 adults had ≥ 2 *ALPL* variants and 103 adults had 1 *ALPL* variant).

Among children and adults who manifested signs and symptoms of HPP after 6 months of age, patients with ≥ 2 *ALPL* variants were more likely to have a history of skeletal, dental, muscular, and neurological manifestations than those with 1 variant [49]. However, patients with 1 variant still had substantial disease burden: over one half of patients with 1 variant reported dental manifestations and pain, one third reported skeletal manifestations, and one quarter reported muscular manifestations. Approximately 9% had neurological manifestations.

Across each of these 3 analyses, median quality-of-life scores among patients in all groups were lower than those of a healthy population [40, 48, 49]. Collectively, data from the registry support that, regardless of age at symptom onset, primary skeletal or nonskeletal symptomology and number of *ALPL* variants, impose a high disease burden and have a substantial impact on quality of life.

### New understanding of the HPP disease continuum and a proposed new classification system

Historically, patients with HPP have been subdivided into various “forms” or “subtypes” based on the age at which the first manifestation of HPP symptoms were observed, including prenatal benign, perinatal, infantile, childhood, and adult HPP [50]. This classification system also includes odonto-HPP, which is characterized by presence of dental manifestations but no other apparent clinical features [50].

This separation into different forms of HPP, which was based on a cross-sectional analysis, does not reflect the longitudinal course of the disease or the fact that disease manifests on a continuum [7, 10, 17, 51]. While some manifestations of HPP depend on the patient’s age (e.g., rickets, which can occur only during childhood), different HPP subtypes or forms are now regarded as a continuum of a single disease in which symptoms can manifest and complications can occur throughout a patient's lifespan [52]. The existing classification system is also limited since a substantial proportion of adults with HPP may have developed the disease in childhood but experienced a delay in receiving the appropriate diagnosis [18]. As such, the existing HPP classification system warrants further evaluation to best align with the most recent scientific evidence.

We propose a new approach to how we think about HPP as a continuum, with nomenclature that reflects the understanding that there is substantial phenotypic overlap and heterogeneity. Many patients do not fit neatly into the existing nosology. In the new classification, patients are divided into 2 main categories: *early-onset HPP* and *late-onset HPP*. Like the previous classification system, this new approach (Table 1) distinguishes patients by age at first disease manifestation, with early-onset defined as patients who present at age < 6 months (with or without respiratory failure) and late-onset defined as patients who present at age ≥ 6 months.

**Table 1:** Comparison of existing and newly proposed HPP disease classifications

Existing Classification [50]	Age of Onset	New Classification
**Prenatal benign**	In utero	Early onset (< 6 months)
**Perinatal** (with or without respiratory failure and/or vitamin B_6_-responsive seizures)	Prenatal to 4 weeks postnatal	
**Infantile** (with or without respiratory failure and/or vitamin B_6_-responsive seizures)	Before 6 months	
**Childhood/juvenile/odonto**	6 months to 18 years	Late onset (≥ 6 months)
**Adult/odonto**	After 18 years	

HPP, hypophosphatasia

This classification is rooted in 2 key observations from the registry, which have broadened our understanding of HPP: (1) that the disease in patients who present before 6 months of age is biologically different from the disease in patients who present after 6 months of age based on the presence of life-threatening manifestations and greater likelihood of having 2 *ALPL* variants and (2) that there are few differences in overall disease burden among patients who present after 6 months of age [13, 40, 43, 48, 49, 53]. Thus, this new classification builds on the existing framework but accounts for our increased understanding of HPP genetics, overlap of symptoms, the evolving nature of the disease over time, and disease prognosis. Similar classifications are used in lysosomal acid lipase deficiency, Pompe disease, and other diseases [54–56].

In addition to the primary categories presented in this classification system, we concur with others [22, 37] on the use of 2 additional terms to describe individuals who have a genetic or a biochemical profile of HPP but no overt clinical features of the disease. Such individuals are often relatives of patients with HPP who were identified by cascade testing or may have been incidentally discovered when serum ALP activity was found to be low. The term *“subclinical HPP”* has been coined to describe clinically asymptomatic individuals with both genetic features (*ALPL* variant and/or positive family history) and the biochemical signature of HPP (i.e., low ALP activity with one or more elevated substrates: PLP, PEA, or PPi). A recent publication described in detail a cohort of 43 healthy individuals without overt clinical features who had an *ALPL* variant and biochemical traits of HPP [57]. In contrast, the term “*asymptomatic carrier*” should be used to describe asymptomatic individuals with genetic features (i.e., presence of an *ALPL* variant) but no biochemical or clinical features. Individuals with subclinical HPP should be monitored periodically for signs of clinical disease manifestation, with the knowledge that such individuals may or may not progress to overt clinical disease. Of note, clinical manifestation needs to be differentiated from the possible presence of histological, occult osteomalacia. Typically, osteomalacia (excessive undermineralized osteoid) is found in bone biopsy samples from patients with HPP [58, 59]. There may well be hidden histological bone hypomineralization present in subclinical HPP, but this theory requires bone biopsy studies to examine.

### Effectiveness and safety of asfotase alfa

Asfotase alfa is an enzyme replacement therapy that treats the underlying cause of HPP: deficient tissue-nonspecific ALP activity. Asfotase alfa was first approved in 2015 for treatment of all patients with HPP (regardless of age at disease onset) in Japan and patients of all ages with pediatric-onset HPP in the United States and European Union [60–62]. The first clinical trials with asfotase alfa were conducted almost exclusively in children with early-onset HPP, and results of the trials showed sustained improvements in survival rate, rickets, respiratory function, and motor function [32, 46, 63–65]. One clinical study assessed asfotase alfa in adolescents and adults with HPP and demonstrated improvements in bone mineralization, pain, and functional ability [58].

In the 10 years after approval of asfotase alfa, analyses of data from patients with late-onset HPP who are enrolled in the registry showed that treatment significantly improved walking ability, pain, and quality of life among adults throughout 3 years of treatment and improved quality of life among children who started treatment after 2 years of age [66, 67]. Injection site reactions were the most common adverse event in analyses of treated patients in the registry [66, 67]. The asfotase alfa (Strensiq, Alexion, AstraZeneca Rare Disease, Boston, MA, USA) US prescribing information lists injection site reactions, lipodystrophy, ectopic calcifications, and hypersensitivity reactions as occurring in ≥ 10% of treated patients [62], although ectopic calcifications are also observed in untreated patients. Hypersensitivity reactions, including anaphylaxis, are also listed in the US prescribing information, although these reactions have not been reported in any of the publications based on data from the registry. These studies have created a foundation for further improvement in the understanding of HPP pathology. The well-characterized repertoire of clinical assessments used to evaluate asfotase alfa will be used in the investigation of efzimfotase alfa, a novel ALP enzyme replacement therapy currently in clinical development [68, 69].

## Conclusions

**Fig. 6:**
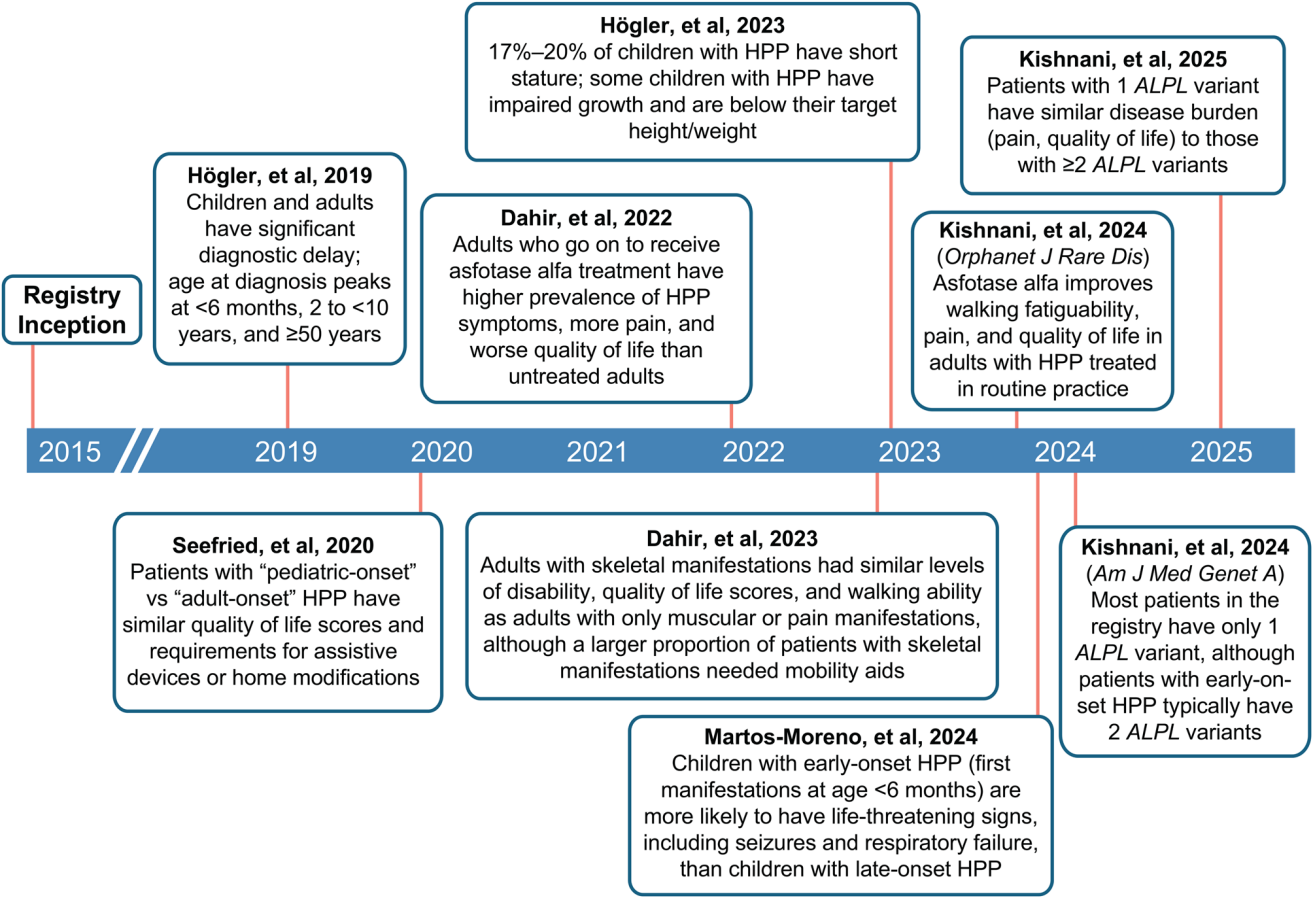
Timeline of registry publications and their major findings. HPP, hypophosphatasia

Learnings from the past 10 years have expanded our understanding of HPP diagnosis, burden, and classification (Fig. 6). Data from the registry have directly helped to overcome challenges related to diagnosis and treatment of HPP by supporting comprehensive assessment of a wide range of clinical manifestations as well as measurement of PLP and PEA in the diagnosis of HPP. These assessments have increased our recognition of multisystem, nonskeletal manifestations and collectively support the notion that HPP is more than a skeletal disorder. These findings may have important implications for future diagnostic criteria, particularly in adults. The high level of disease burden among registry patients regardless of age of onset, presence of skeletal manifestations, or number of *ALPL* variants may be important in determining which patients should receive treatment with enzyme replacement therapy, especially given that symptoms of HPP may evolve and accumulate throughout the course of a patient’s life [9]. However, the high carrier frequency of *ALPL* variants [2, 3] also implies that asymptomatic carriers and individuals with subclinical HPP may be incidentally found when screening for osteoporosis, with a risk of misdiagnosis of HPP particularly among postmenopausal women [57]. The diagnosis of HPP therefore requires more than the combination of a biochemical signature and an *ALPL* variant – this may become increasingly important as genomic sequencing becomes more accessible to patients and the general population. Further research is required to refine diagnostic criteria and to confirm the presence of clinical manifestations of HPP among patients in the registry. Lack of clear, concise guidance on assessment of ALP substrates (i.e., whether fasting is required and how long patients should discontinue vitamin B_6_ supplementation) is another future challenge that must be overcome to accurately diagnose patients.

Findings from the registry have also reshaped our understanding of HPP classification. Our proposed new disease classification supports distinction between patients with early-onset disease (at age < 6 months), who have the most significant phenotype, or late-onset disease (at age ≥ 6 months), as patients in these groups have different disease outcomes and prognoses. The new, simplified classifications alleviate challenges related to recognition of disease burden in adults and removes artificial subdivisions of adult patients into those having pediatric- versus adult-onset disease. Future analyses of the registry will continue to expand upon this growing knowledge and include long-term follow-up of patients to understand natural history of the disease and long-term effectiveness of asfotase alfa and other treatments in development.

## Supplementary Information

Below is the link to the electronic supplementary material.

Supplementary Material 1

## Data Availability

Data are available on reasonable request. Data may be obtained from a third party and are not publicly available. Alexion, AstraZeneca Rare Disease will consider requests for disclosure of clinical study participant-level data provided that participant privacy is assured through methods like data de-identification, pseudonymization or anonymization (as required by applicable law), and if such disclosure was included in the relevant study informed consent form or similar documentation. Qualified academic investigators may request participant-level clinical data and supporting documents (statistical analysis plan and protocol) pertaining to Alexion-sponsored studies. Further details regarding data availability and instructions for requesting information are available in the Alexion Clinical Trials Disclosure and Transparency Policy at https://www.alexionclinicaltrialtransparency.com/data-requests/
