# Supplementary material for: The Global Hypophosphatasia Registry: lessons learned from a decade of real-world data
Source: Orphanet J Rare Dis. 2025 Nov 24;20:626. doi: 10.1186/s13023-025-04129-w (PMC12751868; doi:10.1186/s13023-025-04129-w)
Supplement: Supplementary file 1 — Supplementary Material 1 [file 13023_2025_4129_MOESM1_ESM.pdf]

Supplementary Figure 1. Patient flow diagram.

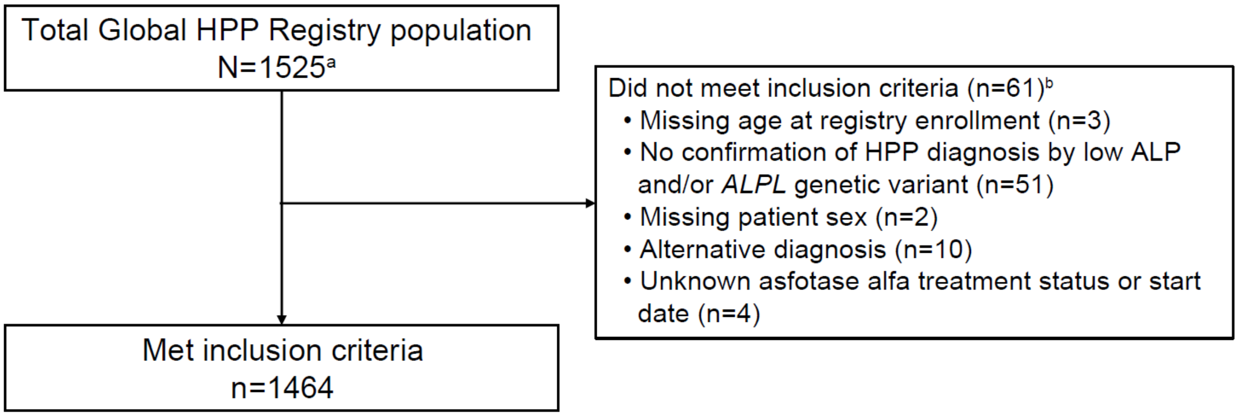

[a]Number of patients enrolled as of December 2024.
[b]Patients may have had multiple reasons for exclusion from study population (categories are not mutually exclusive).
ALP, alkaline phosphatase; HPP, hypophosphatasia.

Supplementary Methods

Patients

The Global HPP Registry (NCT02306720; EUPAS13514) is an ongoing, prospective, observational study that began enrolling patients in 2015. The registry captures data from untreated patients with HPP, as well as from those treated with asfotase alfa enzyme replacement therapy. Data collected in the registry also include HPP natural history and treatment safety and effectiveness. Patients providing data used in the additional analyses presented in this manuscript had the following recorded in the registry: confirmed diagnosis of HPP (low ALP activity and/or presence of *ALPL* variant[s]) without discontinuation from the registry because of an alternative diagnosis; enrollment date; nonmissing sex and age at enrollment/date of birth; and known asfotase alfa treatment status and treatment start date if ever treated. The data cut for this analysis was December 2024.

Age and Sex at Diagnosis

Patterns in the age of diagnosis of patients with HPP were assessed in histograms. Age groups were defined as infancy (0 to <1 year); early childhood (1 to <5 years); middle childhood (5 to <10 years); adolescence (10 to <15 and 15 to <20 years), young adulthood (20 to <40 years); middle adulthood (40 to <65 years) and late adulthood (≥65 years). A curve connecting age groups at time of diagnosis was created using the smooth connect option in SAS Life Science Analytics Framework. A histogram displaying distribution of male and female patients within each age category at diagnosis was also generated.

ALP Substrate Analysis

Concentrations of plasma PLP and urinary PEA were assessed. All available substrate testing data from patients who had never been treated with asfotase alfa were considered for analysis. Among patients who were treated with asfotase alfa, any substrate testing data from prior to asfotase alfa treatment start were considered. The number and percentage of patients with at least one test were summarized by geographic region (United States, Canada, Europe [United Kingdom, France, Germany, Spain, Portugal, Belgium, Italy, Poland, Austria, Greece, and Ireland], Japan, Australia, and other [Israel, Saudi Arabia, Russia, Taiwan, and Turkey]), with the number of patients enrolled in each region as the denominator. For patients with a nonmissing site-entered upper limit of normal (ULN), the percentage of patients with a test result above the ULN at any time were summarized. PLP values were converted to ng/mL, and PEA

values were converted to nmol/mg creatine; results were plotted and additionally summarized as median (min, Q1, Q3, max). Mean values are not reported because the data are skewed and may lead to misinterpretation. Results with missing units or units that could not be converted were excluded from the analyses of the lab distributions. Values that were deemed implausible, likely owing to data entry issues, were excluded. Analyses of substrate testing data were reported by the age at testing (<18 years or ≥18 years of age at the last assessment). Correlations between the most recent treatment-naive ALP activity and ALP substrate value measured on the same day were assessed via Spearman correlation coefficients by age in patients with low ALP at testing (<160 U/L for children <18 years of age at testing and <40 U/L for adults ≥18 years of age at testing). An exponential model fitted using SAS PROC NLIN was used to assess nonlinear relationships.

Statistical Analyses

All statistical analyses were performed with SAS Life Science Analytics Framework, version 5.4.1b (SAS Institute, Cary, NC).
